# Expression and prognostic values of ARID family members in breast cancer

**DOI:** 10.18632/aging.202489

**Published:** 2021-02-11

**Authors:** Jinfeng Zhang, Siyu Hou, Zilong You, Guozheng Li, Shouping Xu, Xianyong Li, Xianyu Zhang, Bo Lei, Da Pang

**Affiliations:** 1 Department of Breast Surgery, Harbin Medical University Cancer Hospital, Harbin, China; 2 Heilongjiang Academy of Medical Sciences, Harbin, China; 3 Department of Critical Care Medicine, The First Affiliated Hospital of Harbin Medical University, Harbin, China

**Keywords:** biomarkers, tumor, breast neoplasms, prognosis

## Abstract

The ARID family is a superfamily of 15 members containing a domain that interacts with AT-rich DNA elements. However, the expression and prognostic roles of each ARID in breast cancer are still elusive.

We used the TCGA and Kaplan-Meier plotter databases to assess the expression and prognostic values of ARID mRNA levels in breast cancer respectively. In the present study, 6 members were significantly lower in tumor tissues than those in the normal tissues, while 6 members were significantly higher. Further assessment of ARID expression in breast cancer with different molecular subtypes, 3 members were significantly higher in no-luminal molecular subtype than those in the luminal molecular subtype, and 6 members were significantly higher. In regard to prognostic values, high expression of ARID1A, ARID2, ARID3B, ARID4A, ARID5A, ARID5B, JARID1A were associated with favorable outcome, while ARID4B and JARID1B were correlated to a worse outcome. We further analyzed the prognostic value of ARID in different intrinsic subtypes and clinicopathological features of breast cancer. We found many meaningful ARID family biomarkers in breast cancer. The relevant results will expound the role of ARID in breast cancer and may further provide new insight to explore the ARID-targeting reagents for treating breast cancer patients.

## INTRODUCTION

Breast cancer is one of the most common malignant tumors among women in the world, and the incidence of breast cancer in 2018 accounted for 24.2% of female cancers [1]. Due to early discovery and multiple treatment modalities, incidence and mortality rates are declining. However, tumor recurrence and distant metastasis are still the chief problems contributing to high mortality [2]. Although some genes have been used for treatment in breast cancer, the clinical efficacy is limited [3]. Thus, novel potential target genes used to predict or treat breast cancer are waiting for discovery.

The ARID (AT-Rich Interaction Domain) family is a superfamily of 15 members containing ARID1A, ARID1B, ARID2, ARID3A, ARID3B, ARID3C, ARID4A, ARID4B, ARID5A, ARID5B, JARID1A, JARID1B, JARID1C, JARID1D and JARID2 [4]. There are 11 genomic loci encoded ARID proteins: JARID1C, JARID1D on sex chromosome Xp11.22 and Yq11.223 respectively, the remaining members on euchromosome (ARID1A on chromosome 1p36.11, ARID1B on chromosome 6q25.3, ARID2 on chromosome 12q12, ARID3A on chromosome 19p13.3, ARID3B on chromosome 15q24.1, ARID3C on chromosome 9p13.3, ARID4A on chromosome 14q23.1, ARID4B on chromosome 1q42.3, ARID5A on chromosome 2q11.2, ARID5B on chromosome 10q21.2, JARID1A on chromosome 12p13.33, JARID1B on chromosome 1q32.1, JARID2 on chromosome 6p22.3). ARID family, as transcription regulators are involved in regulating cellular growth, differentiation, and development in multiple cancers [5]. For example, ARID1A, as a candidate tumor-suppressor gene in breast cancer, inhibited the cell progress, which also increased the sensitivity of drug therapy [6]. Similarly, ARID1B, ARID2, JARID1C could function mainly as tumor suppressors in cancers [7]. However, ARID3A and ARID4B exerted the role of tumor oncogenes [8]. Several genes have shown diverse roles as tumor suppressors and oncogenes depending on the type of cancer cells [9, 10]. Nevertheless, some ARID family members, such as ARID2, ARID5A or ARID3C et al have been rarely studied in breast cancer. The expression and prognostic values of each ARID, especially at the mRNA level in breast cancer are still intricate.

In this study, we assessed ARID mRNA expression in breast cancer tissues and normal tissues by The Cancer Genome Atlas (TCGA datasets, and the prognostic role of each member of ARID mRNA expression in human breast cancer patients by Kaplan-Meier plotter (KM plotter) database. This is the first time to integrate all the members and analyze them. We found many important and meaningful biomarkers that had no reports on breast cancer. The studies provided a good orientation for the following research.

## RESULTS

### Distinct expression of ARID members in breast cancer patients

**Figure 1:**
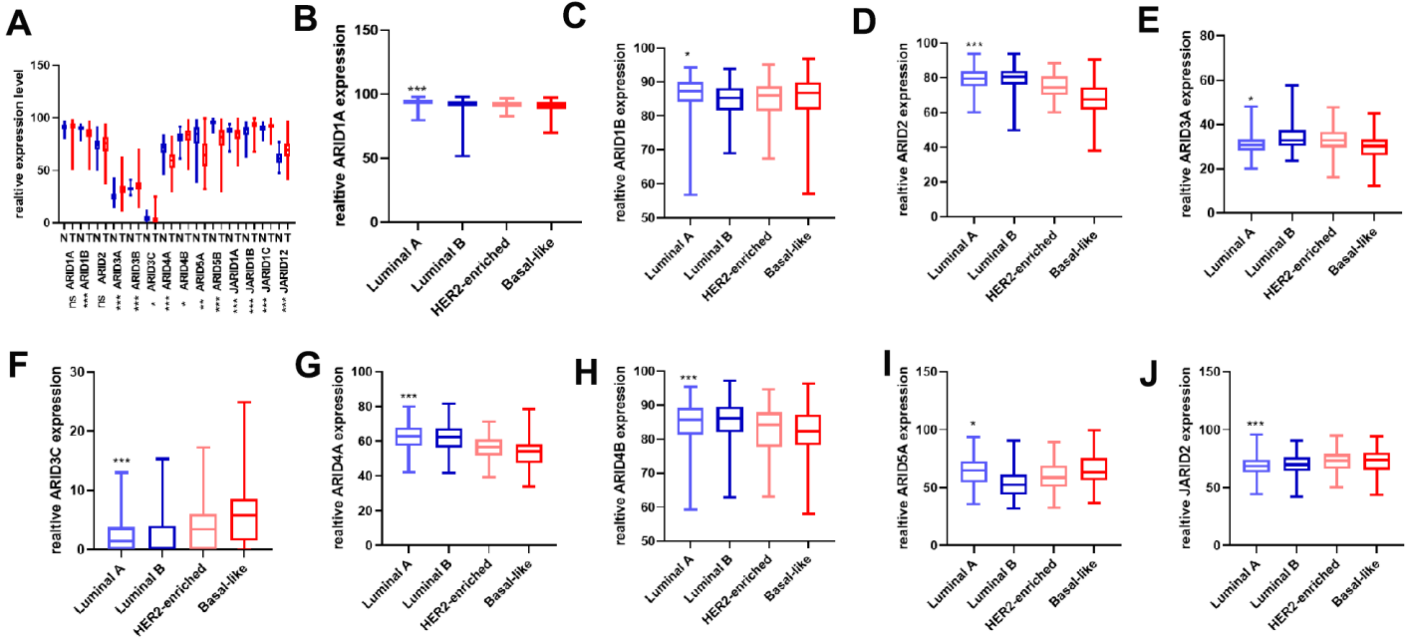
**Distinct expression of ARID members in breast cancer patients.** (**A**) The mRNA expression of all the ARID members was assessed in cancer and normal tissues (*p* values were calculated grouping by Tumor(T)/Normal(N) and using t-test. (**B**–**J**) The mRNA expression of ARID1A, ARID1B, ARID2, ARID3A, ARID3C ARID4A, ARID4B, ARID5A, and JARID2 were assessed in different molecular subtypes(*p* values were calculated grouping by luminal A + luminal B VS. HER2-riched + basal-like and using t-test). (*p < 0.05, **p < 0.01,***p < 0.001).

We analyzed both the tumor tissues and normal breast tissues using the TCGA database. The mRNA expression of ARID3A, ARID3B, ARID4B, JARID1B, JARID1C, JARID2 in tumor tissues was more expressive in normal tissues, ARID1B, ARID3C, ARID4A, ARID5A, ARID5B, JARID1A mRNA expression in tumor tissues were lower than that in the normal tissues (*p*<0.05) (Figure 1A). However, the ARID1A and ARID2 showed these results were not statistically significant. Then, we analyzed and obtained an overall view of the expression of ARID members across different molecular subtypes. The expression of ARID1A, ARID1B, ARID2, ARID3A, ARID4A, and ARID4B in no-luminal subtypes was lower than luminal subtypes, however, ARID3C, ARID5A, and JARID2 mRNA expression were higher in no-luminal subtypes of breast cancer tissues (Figure 1B–1J). The other five genes were not statistically significant (Supplementary Figure 1).

### Prognostic significance of ARID members in all breast cancer patients

**Figure 2:**
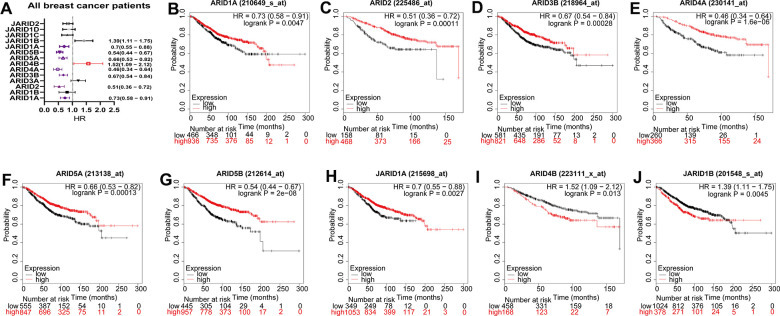
**Prognostic values of ARID members in all breast cancer patients.** (**A**) Prognostic HRs of individual ARID members in all breast cancer. (**B**–**J**) Survival curves of ARID1A(Affymetrix IDs: 210649_s_at), ARID2((Affymetrix IDs: 225486_at), ARID3B(Affymetrix IDs: 218964_at), ARID4A(Affymetrix IDs: 230141_at), ARID5A(Affymetrix IDs: 213138_at), ARID5B(Affymetrix IDs: 212614_at), JARID1A(Affymetrix IDs: 215698_at), ARID4B(Affymetrix IDs: 223111_x_at) and JARID1B(Affymetrix IDs: 201548_s_at).

We respectively assessed the prognostic significances of the mRNA expression of 14 ARID family members (excluding ARID3C, no available matrix data) in human breast cancer patients at https://kmplot.com. Among them, 9 members were correlated with prognosis for all breast cancer patients (Figure 2A). The survival curves were displayed in Figure 2B–2H. High mRNA expression of ARID4B and JARID1B was interrelated with poor survival (Figure 2I, 2J, *p*=0.013 and *p*=0.0045). Low mRNA expression of ARID1A, ARID2, ARID3B, ARID4A, ARID5A, ARID5B, JARID1A were obviously associated with poor OS (Figure 2B–2H, *p*=0.0047, 0.00011, 0.00028, 1.60E-06, 0.0001, 2E−08 and 0.0027 respectively). The mRNA expression quantity of other ARID family members was not associated with OS (Supplementary Figure 2).

### Prognostic significance of ARID members in different breast cancer subtypes

**Figure 3:**
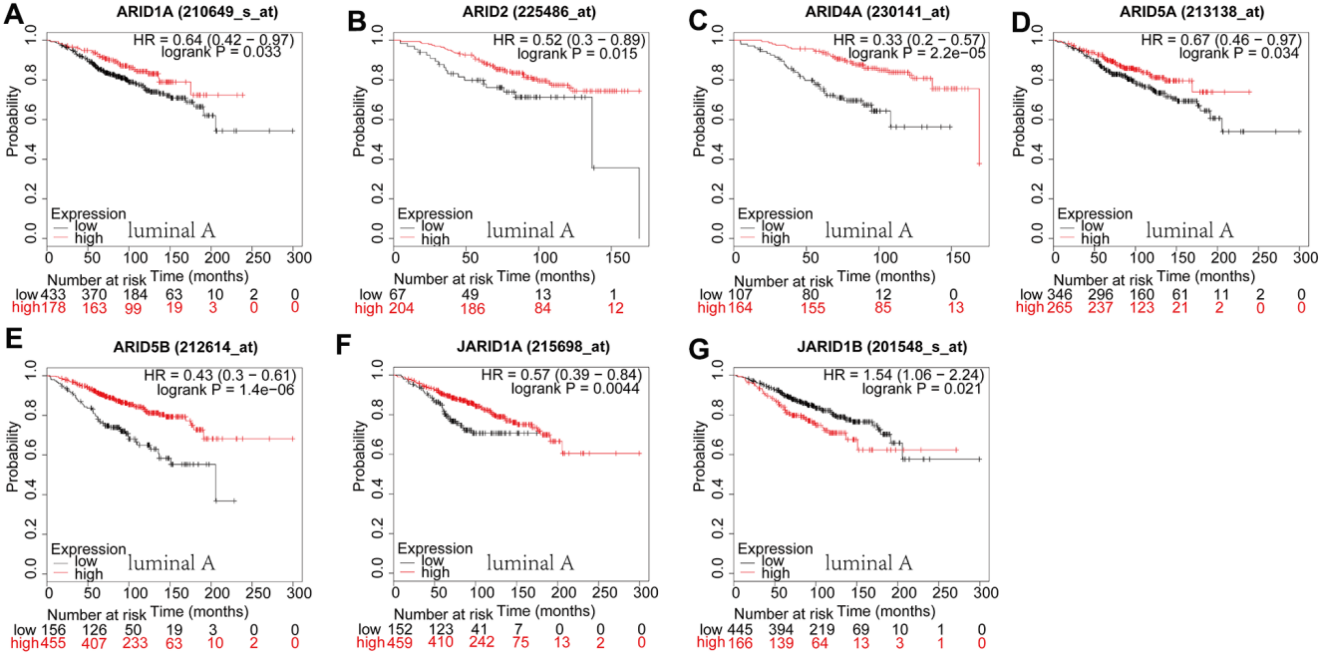
**Prognostic values of ARID members in luminal A type breast cancer patients.** (**A**–**G**) Survival curves of ARID1A(Affymetrix IDs: 210649_s_at), ARID2((Affymetrix IDs:225486_at), ARID4A(Affymetrix IDs: 230141_at), ARID5A(Affymetrix IDs: 213138_at), ARID5B(Affymetrix IDs: 212614_at), JARID1A(Affymetrix IDs: 215698_at) and JARID1B(Affymetrix IDs: 201548_s_at).

Then, we examined the prognostic significance of ARID family members in breast cancer with different intrinsic subtypes, containing luminal A, luminal B, HER2-riched, and basal-like. As shown in Figure 3, For ARID1A (Figure 3A: *p*=0.033), ARID2 (Figure 3B: *p*=0.015), ARID4A (Figure 3C: *p*=2.2e−05), ARID5A (Figure 3D: *p*=0.0338), ARID5B (Figure 3E: *p*=0.0000014) and JARID1A (Figure 3F: *p*=0.0044), their low levels of mRNA expression were correlated with shorter OS in cancers of luminal A type (Figure 3A–3F). For JARID1B (Figure 3G: *p*=0.0209), their high levels of mRNA expression were related to shorter OS in luminal A type of breast cancer patients. The rest of the ARID members were not correlated with OS in luminal A type breast cancer (Supplementary Figure 3).

**Figure 4:**
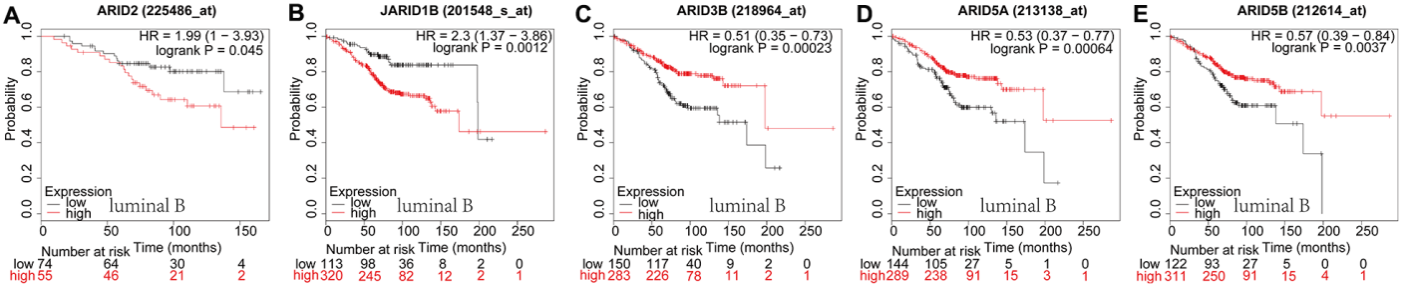
**Prognostic values of ARID members in luminal B type breast cancer patients.** (**A**–**E**) Survival curves of ARID2((Affymetrix IDs:225486_at), JARID1B(Affymetrix IDs: 201548_s_at), ARID3B(Affymetrix IDs: 218964_at), ARID5A(Affymetrix IDs: 213138_at) and ARID5B(Affymetrix IDs: 212614_at).

**Figure 5:**
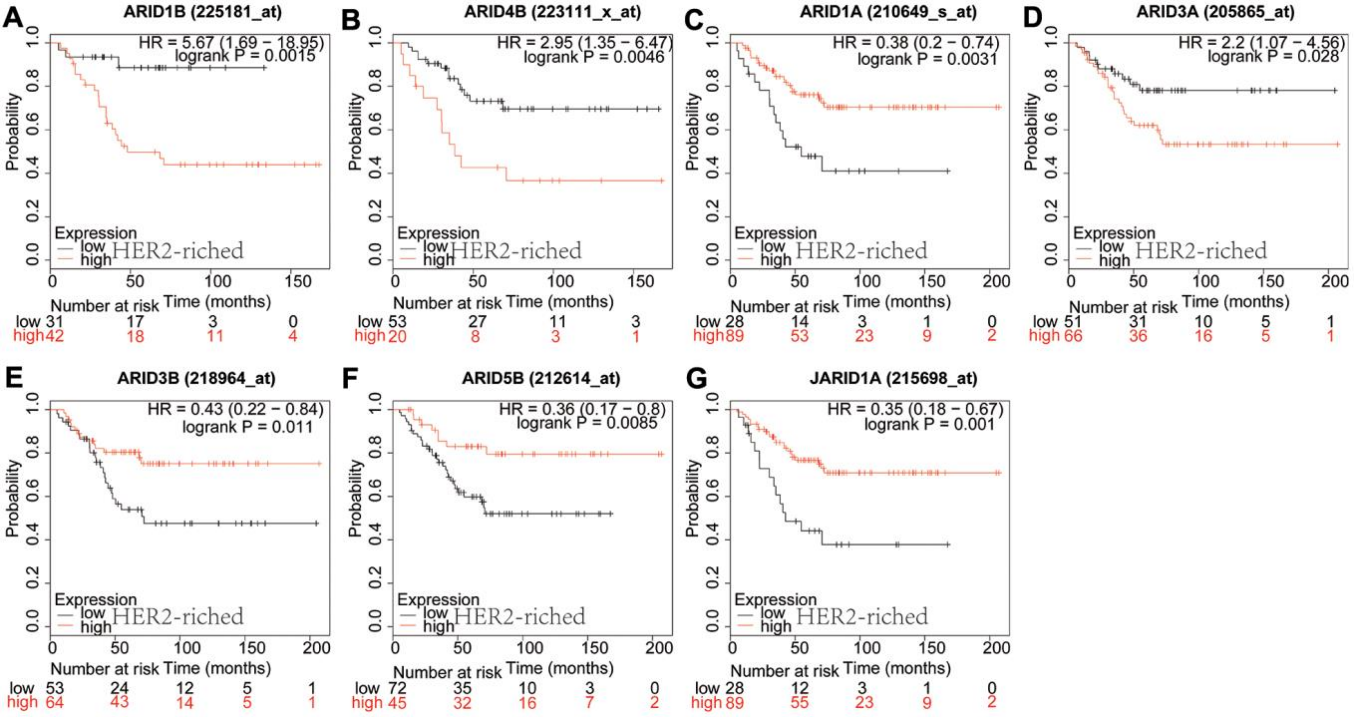
**Prognostic values of ARID members in HER2-riched type breast cancer patients.** (**A**–**G**) Survival curves of ARID1B(Affymetrix IDs: 225181_at), ARID4B(Affymetrix IDs: 223111_x_at), ARID1A(Affymetrix IDs: 210649_s_at), ARID3A(Affymetrix IDs: 205865_at), ARID3B(Affymetrix IDs: 218964_at), ARID5B(Affymetrix IDs: 212614_at) and JARID1A(Affymetrix IDs: 215698_at).

In luminal B type breast cancer, ARID2 (Figure 4A: *p*=0.045) and JARID1B (Figure 4B: *p*=0.0012) were correlated to a shorter OS. However, ARID3B (Figure 4C: *p*=0.0002), ARID5A (Figure 4D: *p*=0.0006) and ARID5B (Figure 4E: *p*=0.0037) were correlated with better survival. The rest members of the ARID family were not related to prognosis in breast cancer of luminal B (Supplementary Figure 4). In HER2-riched breast cancer patients, ARID1B (Figure 5A: *p*=0.0015) and ARID4B (Figure 5B: *p*=0.0046) were interrelated with shorter OS. However, ARID1A (Figure 5C: *p*=0.0031), ARID3A (Figure 5D: *p*=0.0285), ARID3B (Figure 5E: *p*=0.011), ARID5B (Figure 5F: *p*=0.0085) and JARID1A (Figure 5G: *p*=0.001) were associated with better survival. The rest members of the ARID family were not interrelated with prognosis in HER2-riched breast cancer (Supplementary Figure 5).

**Figure 6:**
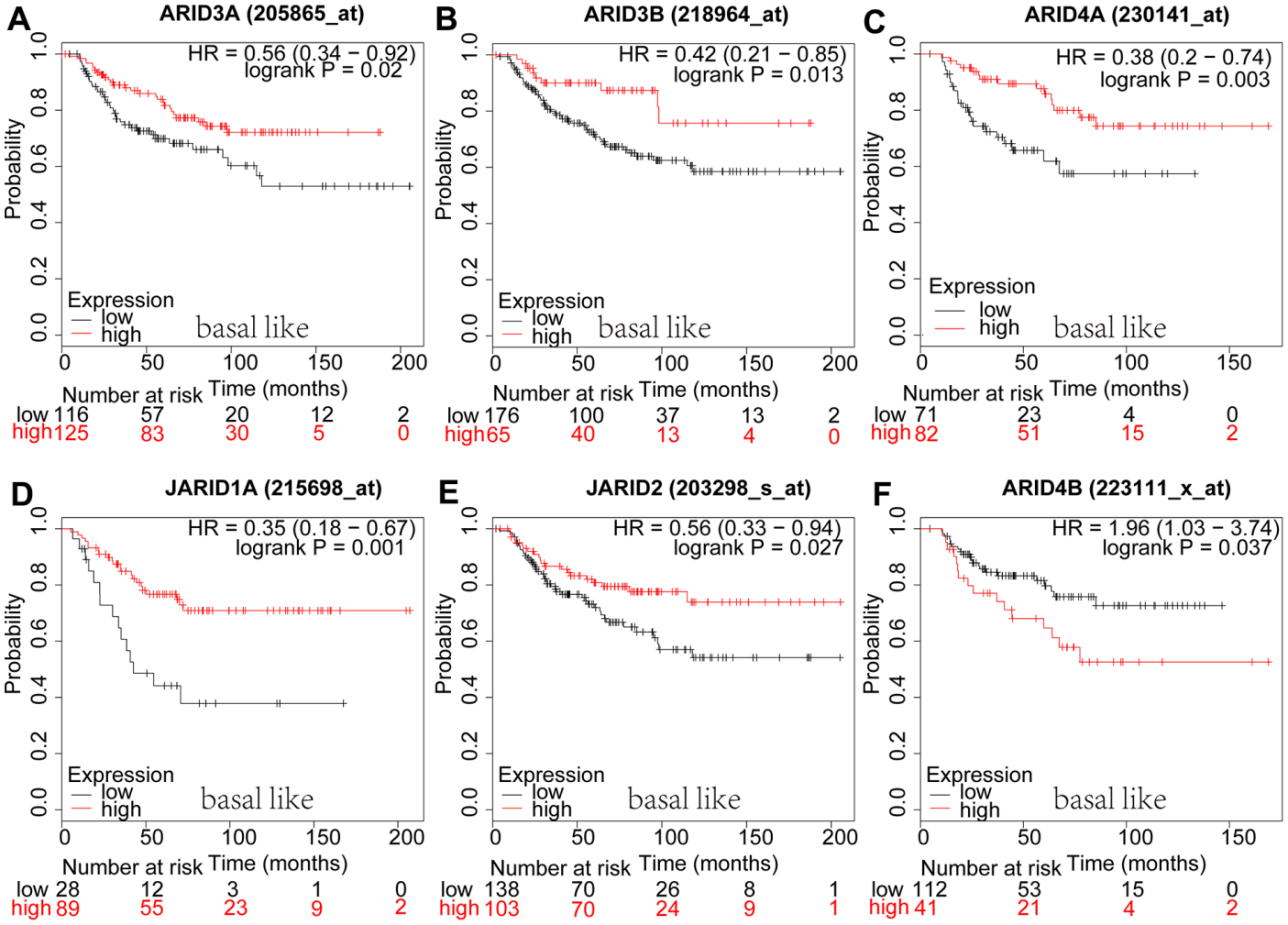
**Prognostic values of ARID members in basal-like type breast cancer patients.** (**A**–**F**) Survival curves of ARID3A(Affymetrix IDs: 205865_at), ARID3B(Affymetrix IDs: 218964_at), ARID4A(Affymetrix IDs: 230141_at), JARID1A(Affymetrix IDs: 215698_at), JARID2(Affymetrix IDs: 203298_s_at), and ARID4B(Affymetrix IDs: 223111_x_at).

In basal-like breast cancer, mRNA expression of ARID4B (Figure 6F: *p*=0.0371) was interrelated with shorter OS. However, ARID3A (Figure 6A: *p*=0.0197), ARID3B (Figure 6B: *p*=0.013), ARID4A (Figure 6C: *p*=0.003), JARID1A (Figure 6D: *p*=0.0265), JARID2 (Figure 6E: *p*=0.0273) were interrelated with better survival. We have discovered the survival curves of the other members of ARID in basal-like breast cancer were irrelevant with prognosis (Supplementary Figure 6).

### Prognostic significance of ARID members in breast cancer patients with different clinicopathological features

Next, we evaluated the relevance of the prognostic values of ARID with other clinicopathological features, obtaining lymph node status, TP53 status and pathological grades. As shown in Table 1, low mRNA expression of ARID5B (*p*=0.045) and JARID1D (*p*=0.0209) was interrelated with worse OS in grade I breast cancer. Low mRNA expression of ARID2 (*p*=0.0035), ARID4A (*p*=0.0349), JARID1A (*p*=0.0473), JARID1D (*p*=0.0322) and high mRNA expression of JARID1B (*p*=0.0067), JARID1C (*p*=0.0407) were interrelated with worse OS in grade II breast cancer patients. Low mRNA expression of ARID1A (*p*=0.0096), ARID2 (*p*=0.0066), ARID3B (*p*= 0.000024), ARID5A (*p*=0.0435), ARID5B (*p*=0.0022), JARID1A (*p*=0.0044), JARID2 (*p*=0.0463) were interrelated with worse OS in grade III breast cancer patients. The high mRNA expression of JARID1B (*p*=0.0061) was discovered to be interrelated to the poor OS in grade III patients.

**Table 1:** Correlation of ARID members with different pathological grade status of breast cancer patients.

**ARID**	**Affymetrix IDs**	**Grades**	**HR**	**95%CI**	**P value**
ARID1A	210649_s_at	I	0.56	0.22 − 1.42	0.2152
		II	0.65	0.42 − 1.01	0.0555
		III	0.64	0.46 − 0.9	**0.0096**
ARID1B	225181_at	I	0.16	0.01 − 1.81	0.0922
		II	0.37	0.1 − 1.4	0.1282
		III	0.71	0.4 − 1.27	0.2507
ARID2	225486_at	I	3.15E+08	0 − Inf	0.26
		II	0.2	0.06-0.66	**0.0035**
		III	0.46	0.26-0.81	**0.0066**
ARID3A	205865_at	I	2.02	0.78 − 5.25	0.1387
		II	1.41	0.92 − 2.17	0.112
		III	1.2	0.87 − 1.67	0.2692
ARID3B	218964_at	I	1.69	0.69 − 4.16	0.2486
		II	1.37	0.87 − 2.16	0.1748
		III	0.49	0.35 − 0.68	**2E-05**
ARID4A	230141_at	I	0	0 − Inf	0.089
		II	0.31	0.1 − 0.98	0.0349
		III	0.4	0.23 − 0.68	**0.0005**
ARID4B	223111_x_at	I	2.54E+08	0 − Inf	0.4026
		II	2.78	0.83 − 9.3	0.0825
		III	1.89	0.97 − 3.68	0.057
ARID5A	213138_at	I	0.42	0.17 − 1.04	0.0534
		II	0.65	0.42 − 1.01	0.0514
		III	0.69	0.49 − 0.99	**0.0435**
ARID5B	212614_at	I	0.38	0.14 − 1.01	**0.045**
		II	0.49	0.31 − 0.75	0.001
		III	0.6	0.43 − 0.84	**0.0022**
JARID1A	215698_at	I	2.21	0.84 − 5.81	0.0984
		II	0.58	0.34 − 1	**0.0473**
		III	0.62	0.44 − 0.86	**0.0044**
JARID1B	201548_s_at	I	0.62	0.24 − 1.64	0.3324
		II	1.84	1.18 − 2.87	**0.0067**
		III	1.62	1.14 − 2.3	**0.0061**
JARID1C	202383_at	I	0.57	0.23 − 1.41	0.2198
		II	1.64	1.02 − 2.64	**0.0407**
		III	0.84	0.59 − 1.2	0.35
JARID1D	206700_s_at	I	0.21	0.05 − 0.9	**0.0209**
		II	0.58	0.35 − 0.96	**0.0322**
		III	1.4	0.96 − 2.02	0.076
JARID2	203298_s_at	I	0.57	0.2 − 1.61	0.284
		II	1.37	0.86 − 2.2	0.18
		III	0.71	0.51 − 1	**0.0463**

As shown in Table 2, the low mRNA expression of ARID1B (*p*=0.0023), ARID2 (*p*=0.0439), ARID4A (*p*=0.0056), ARID4B (*p*=0.0317), ARID5B (*p*=0.00054), JARID1D (*p*=0.043), JARID2 (*p*=0.0068) were interrelated with poor survival in breast cancer patients with negative lymph node. The mRNA expression of ARID1B (*p*=0.0375), ARID4B (*p*=0.0086), JARID1B (*p*=0.0003) and JARID1D (*p*=0.0138) were interrelated with worse survival in breast cancer patients with positive lymph node. The mRNA expression of ARID2 (*p*=0.0043), ARID3A (*p*=0.0423), ARID4A (*p*=0.000052) and ARID5B (*p*=0.0111) were interrelated with much better survival in lymph node positive breast cancer patients.

**Table 2:** Correlation of ARID members with different lymph node status of breast cancer patient.

**ARID family**	**Affymetrix IDs**	**Lymph node status**	**HR**	**95%CI**	**P value**
ARID1A	210649_s_at	negative	0.2523	0.55 − 1.17	0.2523
		positive	0.68	0.45 − 1	0.0507
ARID1B	225181_at	negative	0.25	0.09 − 0.64	**0.0023**
		positive	2.83	1.02 − 7.86	**0.0375**
ARID2	225486_at	negative	0.39	0.15-1.01	**0.0439**
		positive	0.44	0.24-0.78	**0.0043**
ARID3A	205865_at	negative	0.77	0.53 − 1.12	0.1714
		positive	0.61	0.38 − 0.99	**0.0423**
ARID3B	218964_at	negative	0.69	0.47 − 1.01	0.0566
		positive	0.69	0.47 − 1.02	0.0626
ARID4A	230141_at	negative	0.1	0.01 − 0.75	**0.0056**
		positive	0.35	0.2 − 0.59	**5E-05**
ARID4B	223111_x_at	negative	0.36	0.14 − 0.95	**0.0317**
		positive	2.05	1.19 − 3.53	**0.0086**
ARID5A	213138_at	negative	0.72	0.49 − 1.06	0.0927
		positive	0.71	0.46 − 1.09	0.1112
ARID5B	212614_at	negative	0.47	0.32 − 0.68	**5E-05**
		positive	0.6	0.41 − 0.89	**0.0111**
JARID1A	215698_at	negative	0.71	0.46 − 1.09	0.1158
		positive	0.78	0.48 − 1.29	0.3346
JARID1B	201548_s_at	negative	0.78	0.54 − 1.12	0.1774
		positive	2.06	1.38 − 3.08	**0.0003**
JARID1C	202383_at	negative	0.56	0.35 − 0.88	**0.011**
		positive	1.48	0.95 − 2.31	0.0846
JARID1D	206700_s_at	negative	0.61	0.38 − 0.99	**0.043**
		positive	1.88	1.13 − 3.12	**0.0138**
JARID2	203298_s_at	negative	0.6	0.41 − 0.87	**0.0068**
		positive	0.71	0.47 − 1.07	0.1017

Table 3 has shown mRNA expression of ARID4A (*p*=0.0188) was correlated to better OS in mutant-p53-type breast cancer patients. However, reduced expression of ARID1A (*p*=0.0265), ARID5B (*p*=0.0021), JARID1D (*p*=0.0223) and elevated expression of JARID2 (*p*=0.0125) were associated with poor OS in wild-p53-type breast cancer patients.

**Table 3:** Correlation of ARID members with different p53 status of breast cancer patients.

**ARID family**	**Affymetrix IDs**	**P53**	**HR**	**95%CI**	**P value**
ARID1A	210649_s_at	mutant	0.75	0.35 − 1.61	0.4571
		wild	0.48	0.25 − 0.93	**0.0265**
ARID1B	225181_at	mutant	2.73	0.54 − 13.88	0.2103
		wild	0	0	0
ARID2	225486_at	mutant	0.19	0.02-1.56	0.085
		wild	0	0	0
ARID3A	205865_at	mutant	0.51	0.23 − 1.11	0.0846
		wild	1.48	0.74 − 2.94	0.2637
ARID3B	218964_at	mutant	0.64	0.29 − 1.43	0.2768
		wild	1.4	0.71 − 2.76	0.3227
ARID4A	230141_at	mutant	0.12	(0.02 − 0.99	**0.0188**
		wild	0	0	0
ARID4B	223111_x_at	mutant	0.31	0.08-1.18	0.0699
		wild	0	0	0
ARID5A	213138_at	mutant	0.47	0.21 − 1.07	0.0668
		wild	0.58	0.29 − 1.14	0.1085
ARID5B	212614_at	mutant	1.74	0.79 − 3.82	0.1654
		wild	0.38	0.2 − 0.72	**0.0021**
JARID1A	215698_at	mutant	0.51	0.24 − 1.08	0.0714
		wild	1.48	0.78 − 2.83	0.2277
JARID1B	201548_s_at	mutant	1.93	0.89 − 4.16	0.0887
		wild	1.85	0.95 − 3.59	0.066
JARID1C	202383_at	mutant	2.1	0.98 − 4.5	0.0529
		wild	0.59	0.3 − 1.16	0.1221
JARID1D	206700_s_at	wild	0.44	0.21 − 0.91	**0.0223**
		mutant	1.73	0.8 − 3.78	0.16
JARID2	203298_s_at	wild	2.74	1.2 − 6.25	**0.0125**
		mutant	1.83	0.77 − 4.35	0.17

### ARID1A protein is an important prognostic factor in breast cancer patients

**Figure 7:**
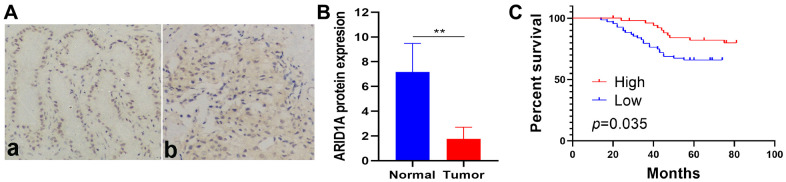
**ARID1A protein expression is low and related to poor survival in breast cancer patients.** (**A**) Representative IHC staining of ARID1A in (**a**) normal tissues (n = 32) and (**b**) tumor tissues (n = 119) (200×). (**B**) The expression of ARID1A protein in tumor tissues was significantly lower than that in adjacent normal breast tissues (p<0.001). (**C**) Low ARID1A expression is associated with poor survival in breast cancer patients, based on a Kaplan–Meier analysis of OS (n=119, log-rank test, p=0.035).

Further to verify the reliability of the above analysis, we chose ARID1A and performed protein level detection and analysis. Immunohistochemical staining (IHC) was used to detect the expression of ARID1A protein in 119 human breast cancer tissues and 32 normal adjacent tissues. A representative image of ARID1A IHC staining is shown in Figure 7A. Compared with normal adjacent tissues, the protein level of ARID1A in cancer tissues was obviously reduced (Figure 7B, *p*<0.01).

Kaplan–Meier OS curve data revealed that the 5-year OS rate of patients with low ARID1A expression was remarkably lower than that of patients with high ARID1A expression (Figure 7C, *p*=0.035). Univariate analysis of OS was performed using Cox regression analysis to determine ARID1A expression (*p*=0.027), Her-2 status (*p*=0.002), Tumor size (*p*=0.026) and LNM (*p*=0.018) as significant prognostic predictors. Multivariate analysis of OS using Cox regression analysis found that Her-2 status (*p*=0.009), tumour size (*p*=0.044), and LNM (*p*=0.028) were independent prognostic factors (Table 4). Current studies have found that low ARID1A expression is related to the poor prognosis of invasive breast cancer. Similar conclusions have also been confirmed in other studies [11].

**Table 4:** Prognostic factors in invasive breast cancer patients using the Cox proportional hazards model.

**Factors**	**Univariate analysis**			**Multivariate analysis**		
	**HR**	**95% CI**	**P-value**	**HR**	**95% CI**	**P-value**
Age (<50/50≤)	0.258	0.224-1.494	0.258	-	-	-
Tumor size (T2+T3/T1)	3.24	1.154-9.098	**0.026**	3.021	1.028-8.875	**0.044**
Lymph node status (+/-)	4.447	1.293-15.482	**0.018**	4.22	1.166-15.273	**0.028**
ER (+/-)	1.118	0.420-2.98	0.823	-	-	-
PR (+/-)	0.782	0.308-1.982	0.604	-	-	-
Her-2 (+/-)	4.21	1.665-10.641	**0.002**	3.625	1.381-9.567	**0.009**
ARID1A(low/high)	3.5	1.151-10.645	**0.027**	2.996	0.943-9.515	**0.063**

## DISCUSSION

ARID family members exert diverse roles in function obtaining development and cell growth regulation [5]. Hence, alterations of the ARID family as members of the chromatin remodeling system contributed to tumorigenic events in various cancers.

Many studies showed ARID1A served a function of tumor suppressor in breast cancer [11]. ARID1A is a key neoplasm suppressor gene that cooperated with CEBPα inhibited UCA1 transcription in breast cancer [12]. Furthermore, ARID1A expression suppressed the accumulation of DNA double-strand breaks caused by radiation and could rescue the loss of radio-resistance triggered by HuR inhibition in patients with breast cancer [13]. In recent research, ARID1A was identified as the chief candidate gene whose depletion determined resistance to the fulvestrant. Resistance to ER degraders appeared in cells and patients of ARID1A inactivation by forwarding a transform from luminal cells to basal-like cells [14]. Moreover, ARID1A expression conferred resistance to several drugs that restrained the HER2/PI3K/mTOR signal in varying degrees [6]. Although there was no mRNA expression difference of ARID1A in breast cancer and normal tissues, the mRNA expression of ARID1A in no-luminal subtypes were lower than those in luminal subtypes of breast cancer tissues, and the prognostic significance of this gene was prominent in the present study. Low ARID1A expression was interrelated with worse survival in all breast cancer patients; we also found the relationship between its mRNA expression and prognosis in luminal A, HER2-riched, grades III and P53-wild breast cancers. Our results consisted of the previous conclusion.

Some studied showed that increased expression of ARID1B forebodes poor survival in triple-negative breast cancer patients [15], while, another one reported ARID1B potentially served as a valuable prognostic indicator and therapeutic target in breast cancer patients with triple-negative subtypes [16]. A promotional role of ARID4B containing cell lines, animal models, and patients had been observed in the progression of metastatic breast cancer by interactions with chromatin-modifying complexes [8]. Here, ARID1B mRNA expression was associated with poor prognosis in HER2-riched breast cancers and lymph node-positive patients, ARID4B mRNA expression, which was higher in breast cancer tissues, was interrelated with unfavorable prognosis in all breast cancer patients. So the effect of ARID1B and ARID4B in breast cancer served as oncogenes in the previous and present study.

ARID4A acted double roles in cancer progression. Down-regulation of ARID4A directly regulated by microRNA-30d promoted tumor progression and in patients with prostate cancer [17]. However, a peptide epitope equal to ARID4A (KASIFLK), which preferentially expresses in breast cancer cell lines and tissues, maybe indicates ARID4A as a tumor oncogene [18]. In contrast to the study, ARID4A was a lower expression in breast cancer tissues than normal tissues, its mRNA expression foreboded better OS in breast cancer patients, peculiarly in luminal A and basal-like type breast cancer, so we spectated ARID4A as a tumor suppressor in breast cancer. Besides, ARID4A acted remarkably tumor suppression in P53-mutant breast cancer patients, so the loss and tumor suppression of ARID4A may be related to the P53 gene in breast cancer.

Some studies revealed frequent inactivating mutations in ARID2 in unstable colorectal cancer and non-small cell lung carcinoma [19], Loss of ARID2 expression also existed during the progression of gastric cancer and hepatocellular [20], ARID2 took part in the important pathway genes in pancreatic cancer too [21]. All the researches showed consistently ARID2 the function of a tumor suppressor. For all I know, so far there are no reports about the association between ARID2 and breast cancer. Our research demonstrated low ARID2 expression was frequent in no-luminal breast cancer type and forecasted poor survival in all breast cancer patients and ER+ breast cancer patients by K-M plotter analysis. ARID3A protein was mainly expressed in B-lymphocytes [22]. Date analysis showed ARID3A might take part in the breast cancer regulatory networks, no other deeper research [23]. Based on our results, high mRNA expression of ARID3A was interrelated to poor prognosis in HER2-riched type breast cancer, but a better prognosis in basal-like type breast cancer.

Concerning ARID3C and ARID5A, there were no studies carried on the relationship between them and cancers as yet. In our study, expression of ARID3C and ARID5A were lower in tumor tissues than in normal tissues. K-M plotter analysis showed that low mRNA expression of ARID5A was obviously related with poor OS in luminal type and all breast cancer patients. In past studies, ARID5A was involved in the inflammatory process [24]. ARID5A plays a vital part in location of cells by translocation from nucleus to cytoplasm under diverse physiological conditions. ARID5A in Cytoplasm elevated the half-time of mRNA, whereas ARID5A in nuclear interacted with TFs, controlled cell proliferation and differentiation [24]. Combining our results, we speculated ARID5A would be a good candidate gene as a tumor suppressor in breast cancer. Like ARID5A, mRNA expression of ARID5B was interrelated with better survival in our study, which indicated the function of a tumor suppressor. However, other another study reported that ARID5B mainly give play to the role of an oncogene in T-cell leukemogenesis by activating the oncogenic transcriptional program [25]. Maybe the results indicated mRNA and protein level expression of ARID5B are functionally demarcative in breast cancer. The role of ARID3B in cancers was controversial. ARID3B, as a target of miR-125a, accelerates the migration of breast cancer and invasion of ovarian cancer, which inclines to play as an oncogene [26]. Furthermore, nuclear expression of ARID3B was positively related to ER status and negatively correlated with ERBB2 status, tumor grade and mitotic index in the breast cancer patients [27]. Nevertheless, a study showed ARID3B and Mycn cooperated in the proliferation and death of mouse stem cells, a ARID3B increased the survival time of cells and Mycn drove the progression of cell cycle [28].

Several studies have revealed that JARID1A and JARID1B give play to the diverse function of oncogenes and tumor suppressors resting with the type of tumor cells. Both JARID1A and JARID1B activated by H3K4 demethylation may play the function of tumor inhibition by enhancing cellular senescence in lung cancer [9]. JARID1B in MCF-7 and MDA-MB-231 of breast cancer cells inhibited cell angiogenesis and invasion by suppressing CCL14 expression [29]. By comparison, several studies indicated that JARID1A and JARID1B were related to tumor progression instead of in inhibition. The depletion of JARID1A suppresses migration, invasion, proliferation, and metastasis of lung cancer, which suggests JARID1A’s oncogenic roles in lung cancer progression [30]. A study reported JARID1B is extensively expressed in estrogen receptor positive breast cancer cell lines and tissues, and interaction with ERα. JARID1B’s low expression in MCF-7 cells brought about an obvious decrease in E2 activated tumor growth in nude mouse. The result demonstrated JARID1B exerted a precise oncogenic role in estrogen-induced growth of ER+ breast cancer [31]. In our study, mRNA expression of JARID1A was lower in breast cancer tissues, and its down-regulated mRNA expression was obviously related to worse OS in all breast cancer patients, notably in luminal A type, HER2-riched and basal-like type breast cancer patients, which acted the function of tumor suppression. While, mRNA expression of JARID1B, which was the higher expression in breast tumor tissues and appeared as an oncogene, was distinctly relevant to worse OS in all breast cancer patients, especially in ER/PR+ breast cancer patients.

To sum up, we analyzed the expression and prognostic values of ARID members’ mRNA expression in breast tumor patients by the TCGA and KM plotter database. Including them, 6 members were meaningfully lower in tumor tissues than those in the normal tissues, 6 members were distinctly higher in cancer tissues than those in the normal mammary gland. Further assessment of ARID expression in breast cancer with different molecular subtypes, 3 members were significantly higher in no-luminal molecular subtype than those in the luminal molecular subtype, and 6 members were obviously higher in luminal molecular subtype than those in the no-luminal molecular subtype. The mRNA expression levels of 7 ARID family members were interrelated to better OS in all breast cancer patients, and 2 members were correlated with poor OS. We further analyzed the expression of ARID1A from the protein level and got similar conclusions.

We found many important and meaningful ARID family biomarkers, and many members had no reports on breast cancer. Our research affords new insights in regard to the function of ARID members to breast tumor progression, which may be helpful for the further discovery of medicine targeting ARID for breast tumor treatment.

## MATERIALS AND METHODS

Normalized gene expression data for 1095 primary breast cancers and 113 normal breast tissues, assayed by RNA-sequencing, was gained from the TCGA data analysis website (http://www.cbioportal.org/index.do). The intergroup difference was evaluated using a t-test. The relevance of mRNA expression of ARID family members to OS was assessed on an network database (https://kmplot.com), which was constituted from Gene Expression Omnibus(GEO) [32]. Survival curves of ARID1A protein expression were plotted using the Kaplan–Meier method, the effect of different variables on overall survival was evaluated using the Cox regression analyses. *p*-value<0.05 was considered to be statistically significant.(*p<0.05, **p<0.01, ***p<0.001).

The expression of ARID1A protein was performed by Immunohistochemistry staining(IHC). Each tissue section was incubated with ARID1A mouse polyclonal antibody solution (SC-32761, Santa Cruz, CA, USA; 1:200 dilution). The staining scoring was calculated by intensity*percentage of positive cells. The IHC staining intensity was graded as 0(no staining), 1 (weak staining), 2 (moderate staining), and 3 (strong staining). The positive staining tumor cells proportion in a field was calculated as 0 (<5%), 1 (5–20%), 2 (20–50%), 3 (50–75%), and 4(> 75%).

## Supplementary Material

Supplementary Figures
