# Supplementary material for: Expression and prognostic values of ARID family members in breast cancer
Source: Aging (Albany NY). 2021 Feb 11;13(4):5621–37. doi: 10.18632/aging.202489 (PMC7950271; doi:10.18632/aging.202489)
Supplement: Supplementary Figures [file aging-13-202489-s001.pdf]

## SUPPLEMENTARY FIGURES

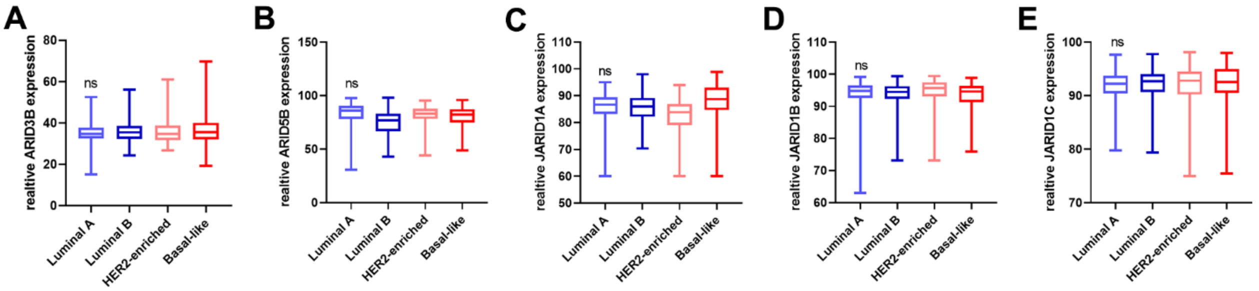


**Supplementary Figure 1. Distinct expression of ARID members in different breast cancer molecular subtypes.** (**A**–**E**) The mRNA expression of ARID3B, ARID5B, JARID1A, JARID1B, JARID1C were assessed in different molecular subtypes (p values were calculated grouping by luminal A + luminal B VS. HER2-riched + basal-like and using t-test).

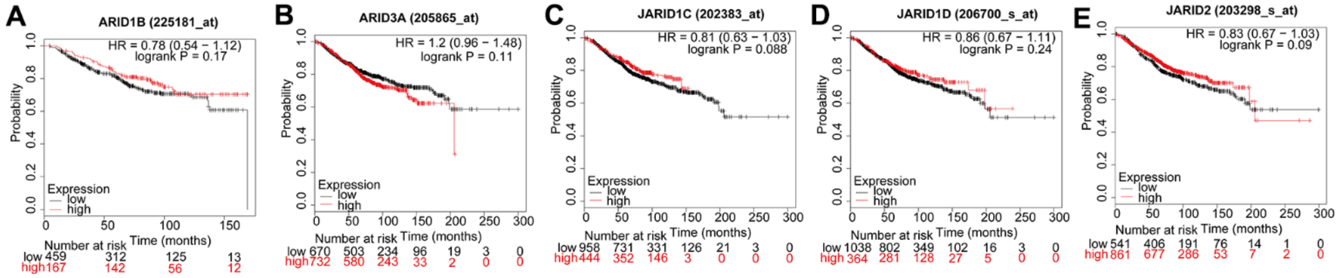


**Supplementary Figure 2. Prognostic values of ARID members in all breast cancer patients.** (**A**–**E**) Survival curves of ARID1B(Affymetrix IDs: 225181_at), ARID3A(Affymetrix IDs: 205865_at), JARID1C(Affymetrix IDs: 202383_at), JARID1D(Affymetrix IDs: 206700_s_at), JARID2(Affymetrix IDs: 203298_s_at).

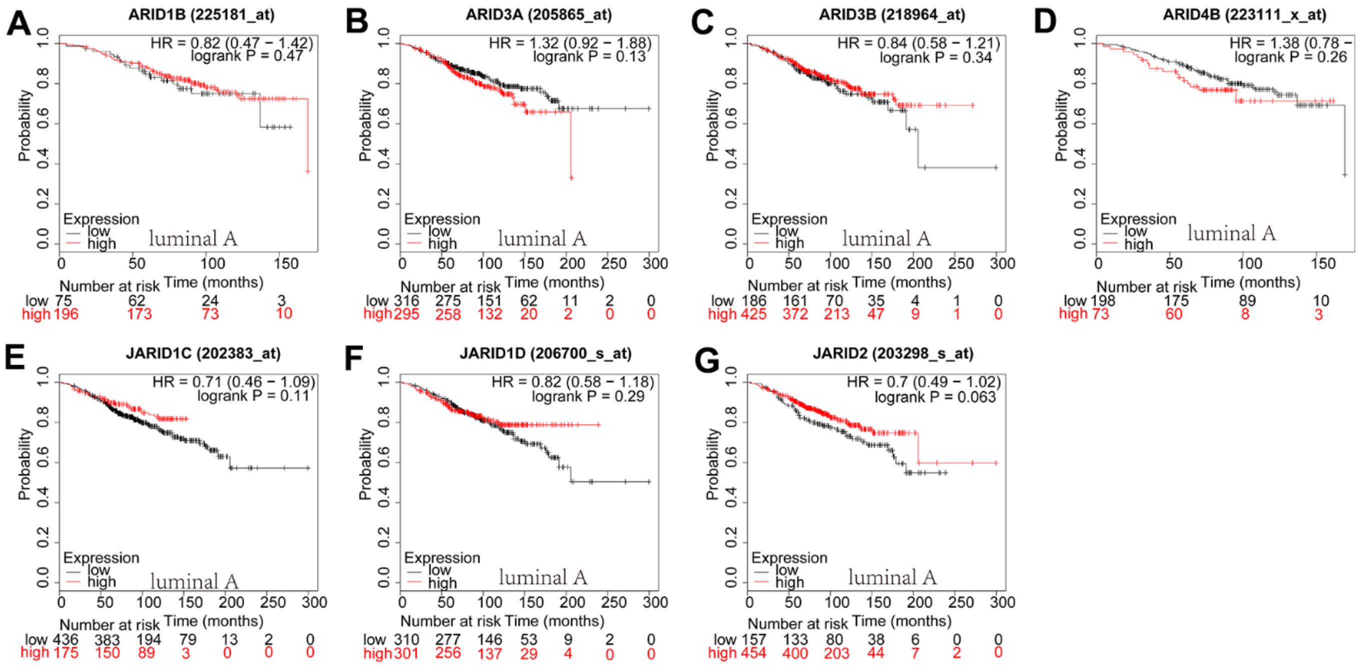


**Supplementary Figure 3. Prognostic values of ARID members in luminal A type breast cancer patients.** (**A**–**G**) Survival curves of ARID1B(Affymetrix IDs: 225181_at), ARID3A(Affymetrix IDs: 205865_at), ARID3B(Affymetrix IDs: 218964_at), ARID4B(Affymetrix IDs: 223111_x_at), JARID1C(Affymetrix IDs: 202383_at), JARID1D(Affymetrix IDs: 206700_s_at), JARID2(Affymetrix IDs: 203298_s_at).

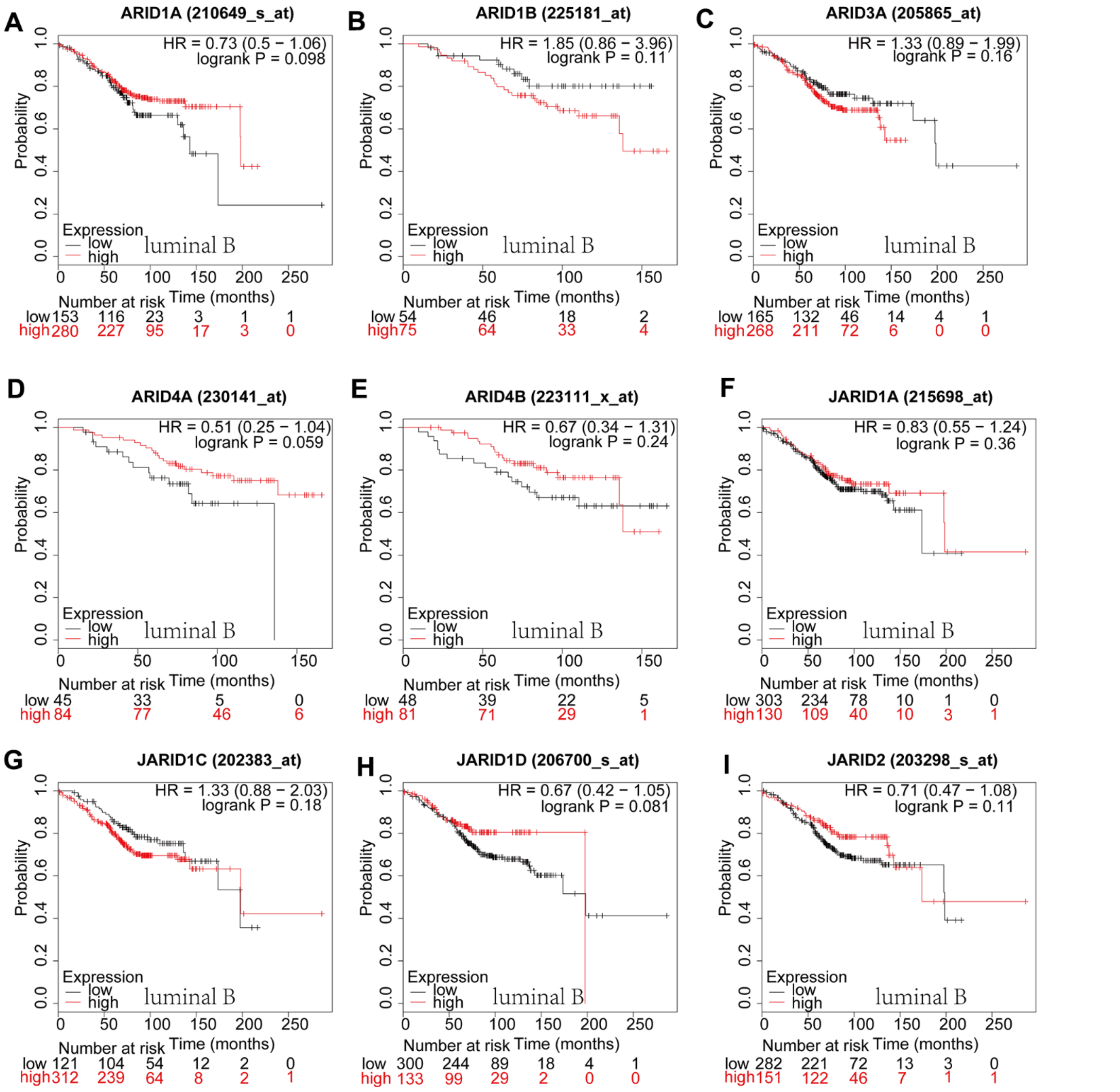


**Supplementary Figure 4. Prognostic values of ARID members in luminal B type breast cancer patients.** (**A**–**I**) Survival curves of ARID1A(Affymetrix IDs: 210649_s_at), ARID1B(Affymetrix IDs: 225181_at), ARID3A(Affymetrix IDs: 205865_at), ARID4A(Affymetrix IDs: 230141_at), ARID4B(Affymetrix IDs: 223111_x_at), JARID1A(Affymetrix IDs: 215698_at), JARID1C(Affymetrix IDs: 202383_at), JARID1D(Affymetrix IDs: 206700_s_at), JARID2(Affymetrix IDs: 203298_s_at).

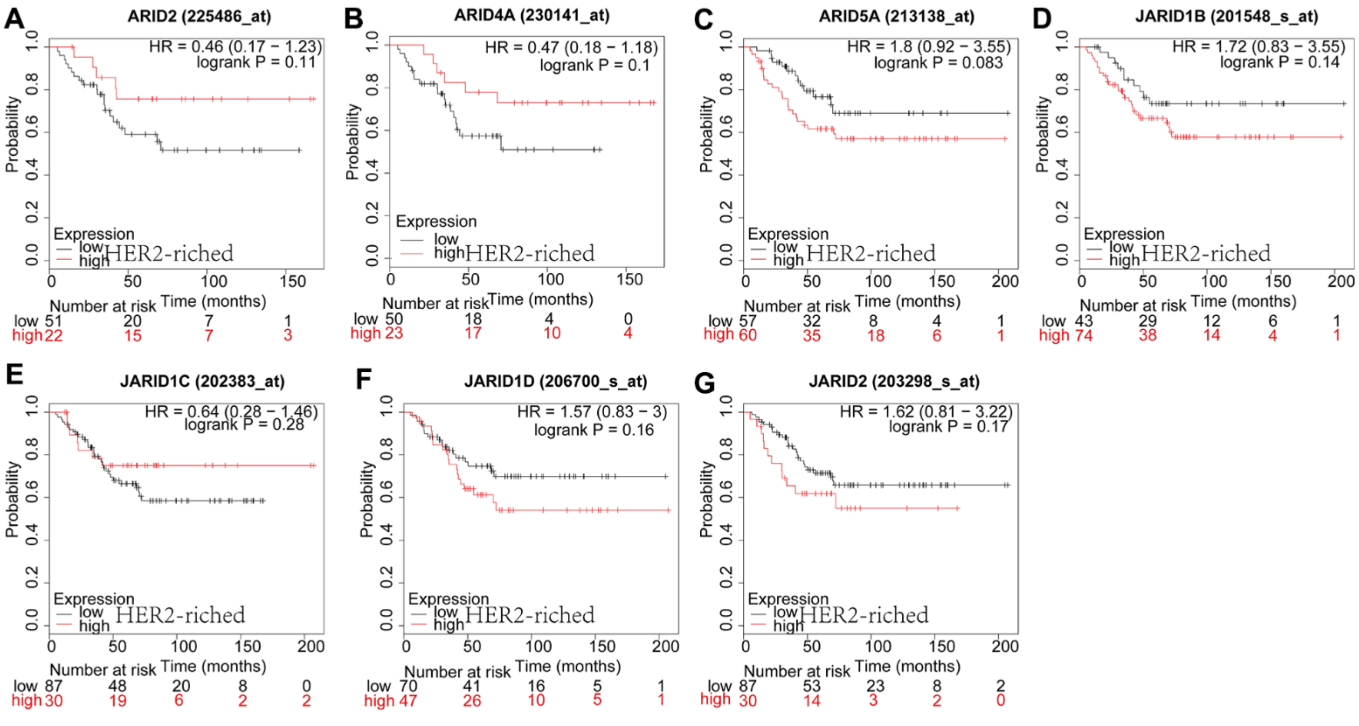


**Supplementary Figure 5. Prognostic values of ARID members in HER2-riched type breast cancer patients.** (**A**–**G**) Survival curves of ARID2((Affymetrix IDs: 225486_at), ARID4A(Affymetrix IDs: 230141_at), ARID5A(Affymetrix IDs: 213138_at), JARID1B(Affymetrix IDs: 201548_s_at), JARID1C(Affymetrix IDs: 202383_at), JARID1D(Affymetrix IDs: 206700_s_at), JARID2(Affymetrix IDs: 203298_s_at).

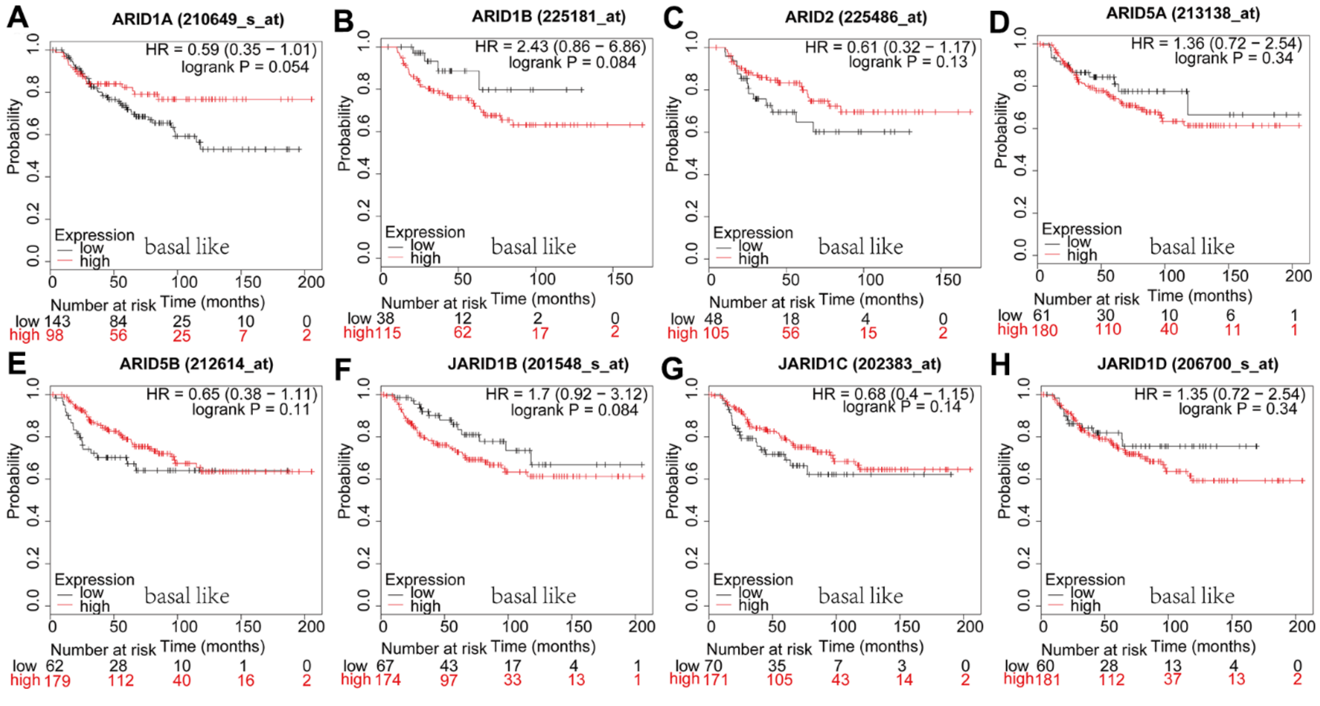


**Supplementary Figure 6. Prognostic values of ARID members in basal-like type breast cancer patients.** (**A**–**H**) Survival curves of ARID1A(Affymetrix IDs: 210649_s_at), ARID1B(Affymetrix IDs: 225181_at), ARID2((Affymetrix IDs: 225486_at), ARID5A(Affymetrix IDs: 213138_at), ARID5B(Affymetrix IDs: 212614_at), JARID1B(Affymetrix IDs: 201548_s_at), JARID1C(Affymetrix IDs: 202383_at), JARID1D(Affymetrix IDs: 206700_s_at).
